# Persistent metabolic acidosis and frailty trajectories after kidney transplantation: a prospective cohort study

**DOI:** 10.1007/s10157-026-02920-y

**Published:** 2026-07-27

**Authors:** Lucian Siriteanu, Andreea Covic, Călin Namolovan, Simona Mihaela Hogaș, Luminița Voroneanu, Adrian Covic

**Affiliations:** 1 Grigore T. Popa University of Medicine and Pharmacy, Iași, Romania; 2 Dr. C. I. Parhon Hospital, Carol I Boulevard No. 50, 700503 Iași, Romania

**Keywords:** Metabolic acidosis, Kidney transplantation, Frailty, Sarcopenia, Serum bicarbonate, Physical function

## Abstract

**Background:**

Metabolic acidosis is common after kidney transplantation and has been linked to adverse graft outcomes, but its potential contribution to functional decline has not been prospectively evaluated. We investigated whether persistent metabolic acidosis is associated with longitudinal frailty trajectories in kidney transplant recipients.

**Methods:**

In this prospective single-center cohort study, 239 adult kidney transplant recipients were followed for 24 months. Serum bicarbonate was measured at baseline, 6, 12, and 24 months, and acidosis persistence was defined by the number of visits with bicarbonate < 22 mmol/L (none, transient, recurrent, or persistent). Frailty was assessed using the Fried phenotype at baseline, 12 months, and 24 months. Longitudinal frailty trajectories were analyzed using mixed-effects ordinal logistic regression.

**Results:**

Frailty prevalence at 24 months increased progressively with greater acidosis persistence, from 7.4% in patients without acidosis to 27.4% among those with persistent acidosis (p for trend = 0.004). In mixed-effects models, increasing acidosis persistence was associated with higher odds of worse frailty category (unadjusted proportional OR [pOR] per category 1.91, 95% CI 1.42–2.56). This association remained significant after adjustment for demographic, transplant-related, and clinical variables (pOR 1.52, 95% CI 1.12–2.06) and after additional adjustment for time-varying kidney function (pOR 1.42, 95% CI 1.04–1.95). Persistent acidosis was also associated with higher frailty scores in linear mixed models (β 0.31 per category increase, *p* < 0.001).

**Conclusion:**

Persistent metabolic acidosis was associated with worsening frailty trajectories after kidney transplantation, suggesting that persistent low bicarbonate may represent a clinically relevant marker of functional decline, although causality cannot be established.

**Supplementary Information:**

The online version contains supplementary material available at https://doi.org/10.1007/s10157-026-02920-y.

## Introduction

Kidney transplantation is the preferred modality of kidney replacement therapy for patients with end-stage kidney disease, offering superior survival and improved quality of life compared with patients receiving maintenance hemodialysis [1, 2]. Frailty is a clinical syndrome characterized by diminished physiological reserve and increased vulnerability to stressors, with reported prevalence rates of up to 20% among kidney transplant recipients [3, 4]. Although a substantial proportion of patients improve during the first post-transplant year, frailty may persist or develop during longer follow-up, underscoring the need for ongoing monitoring of frailty in this population [5–8]. Frailty has been associated with multiple adverse outcomes after kidney transplantation, including an approximately twofold higher risk of mortality and a greater burden of hospitalizations, characterized by increased early readmissions and longer hospital stays. Moreover, frailty has been associated with an increased risk of death-censored graft failure and inferior long-term graft survival following kidney transplantation [7, 9–11].

Metabolic acidosis is a frequent metabolic complication after kidney transplantation, affecting more than one-third of recipients and often occurring earlier than in non-transplant patients with chronic kidney disease (CKD) [12, 13]. In chronic kidney disease, metabolic acidosis has been associated with clinical manifestations resembling physical frailty, including malnutrition, falls, and fractures [14].

Experimental and clinical evidence indicates that metabolic acidosis promotes skeletal muscle wasting, mitochondrial dysfunction, and inflammation—key biological pathways implicated in the development of frailty. Importantly, correcting acidosis improves muscle mass and physical performance, suggesting a potentially modifiable role in physical frailty [15–18].

Despite the biological plausibility linking metabolic acidosis to skeletal muscle dysfunction and frailty, no prospective studies have examined whether persistent metabolic acidosis influences longitudinal frailty trajectories after kidney transplantation. Most studies of post-transplant metabolic acidosis have focused primarily on graft function and mortality, while its potential contribution to functional decline and frailty has received little attention. Given that frailty is a major determinant of long-term outcomes in transplant recipients, identifying modifiable metabolic contributors may have important clinical implications. Therefore, we conducted a prospective cohort study to investigate whether persistent metabolic acidosis is associated with longitudinal frailty trajectories in kidney transplant recipients.

## Materials and methods

### Study design and participants

**Fig. 1:**
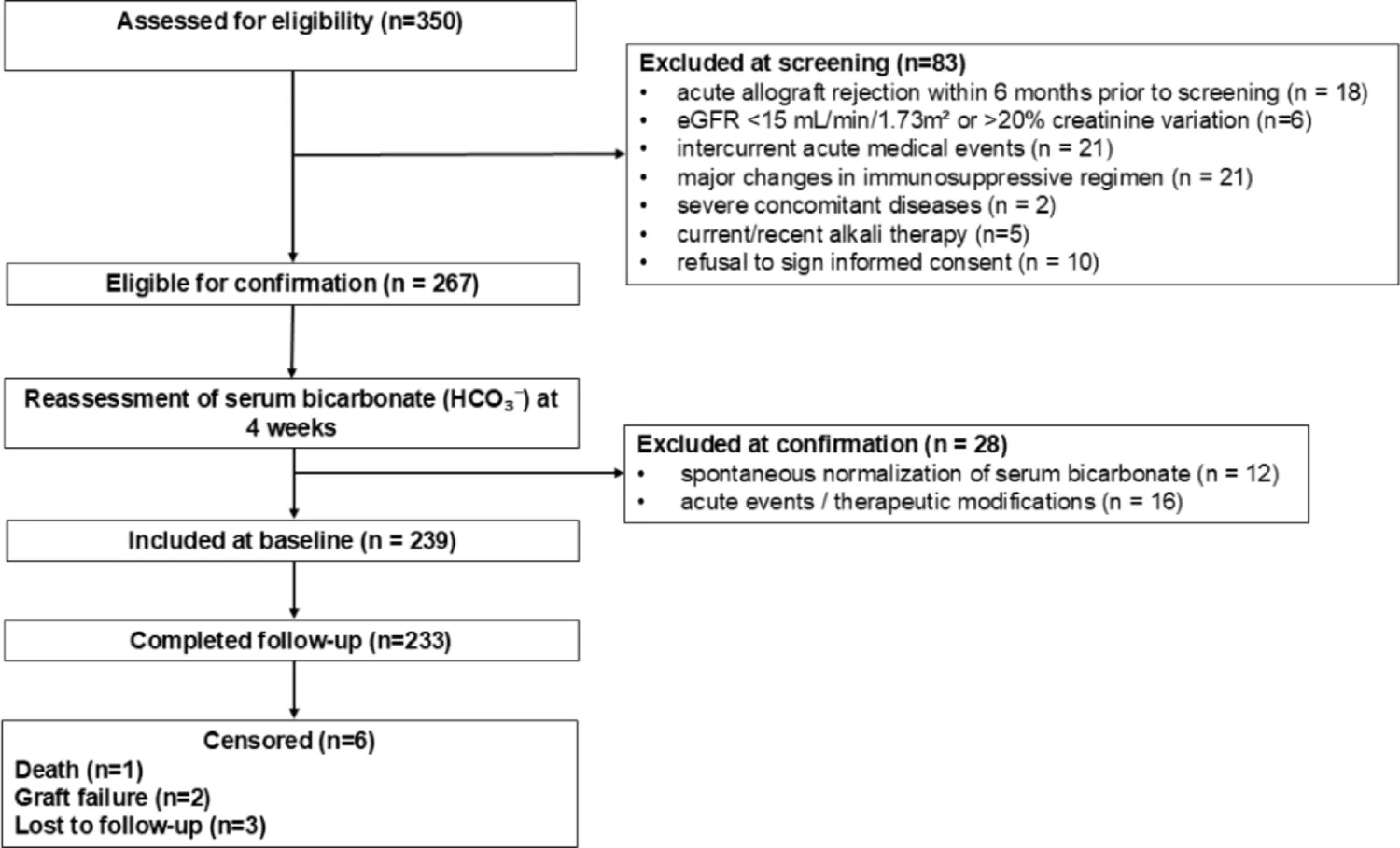
Flow diagram of patient screening, eligibility assessment, and follow-up in the study cohort

We conducted a single-center prospective observational cohort study at the Renal Transplant Unit of “Dr. C. I. Parhon” Clinical Hospital, Iași, Romania. Consecutive adult kidney transplant recipients (≥ 18 years) attending routine outpatient follow-up were screened for eligibility. The patient selection process is illustrated in Fig. 1.

Participants were eligible if they had a functioning kidney allograft ≥ 6 months after transplantation, stable graft function defined as estimated glomerular filtration rate (eGFR) ≥ 15 mL/min/1.73 m² (CKD-EPI 2021 equation) with < 20% variation in serum creatinine over the preceding 3 months, absence of new-onset significant proteinuria with urine protein-to-creatinine (UPCR) ratio ≥ 1000 mg/g or > 50% increase from prior value, and a stable immunosuppressive regimen for at least 3 months (no change in therapeutic class and < 25% dose adjustment). Participants were required to complete follow-up and provide informed consent.

Patients were excluded if they had biopsy-proven or clinically treated acute rejection within the previous 6 months, major immunosuppressive modification within the previous 3 months, or acute intercurrent illness at screening (including active infection requiring antimicrobial therapy, gastrointestinal losses, acute cardiac decompensation, or sepsis). Additional exclusion criteria included severe comorbidities affecting acid–base balance (New York Heart Association [NYHA] class IV heart failure, chronic obstructive pulmonary disease [COPD] with hypercapnia, decompensated cirrhosis, or uncontrolled diabetes mellitus), recent use of medications known to alter acid–base homeostasis (high-dose loop diuretics > 80 mg/day furosemide equivalent, carbonic anhydrase inhibitors, or topiramate within 4 weeks), pregnancy or breastfeeding.

### Exposure: metabolic acidosis and persistence classification

Serum bicarbonate was measured at screening and reassessed 4 weeks later to confirm acid–base status (baseline visit, day 28 ± 7). Metabolic acidosis was defined as serum bicarbonate < 22 mmol/L. The threshold of 22 mmol/L was selected based on the KDIGO 2024 CKD guideline recommendation for initiating alkali therapy [19]. Thereafter, bicarbonate was measured at 6, 12, and 24 months, resulting in four assessments per participant (baseline, 6, 12, and 24 months).

The primary exposure variable was acidosis persistence, defined as the cumulative number of visits with bicarbonate < 22 mmol/L across these four time points. Participants were classified into four categories: no acidosis (0 visits), transient acidosis (1 visit), recurrent acidosis (2 visits), and persistent acidosis (≥ 3 visits). This classification captured the longitudinal pattern of serum bicarbonate and was intended to reflect cumulative metabolic acid exposure during follow-up.

### Follow-up and data collection

Participants were followed prospectively for 24 months from the baseline visit, with scheduled assessments at approximately 6, 12, and 24 months as part of routine outpatient transplant care. Follow-up was censored at death, graft failure, loss to follow-up, or completion of the study period.

Baseline data included demographic characteristics (age, sex), transplant-related variables (time since transplantation, donor type, and immunosuppressive regimen), comorbidity burden assessed using the Charlson Comorbidity Index (CCI), and concomitant medications.

At each visit, laboratory measurements included serum creatinine, estimated glomerular filtration rate (eGFR; CKD-EPI 2021 equation), serum bicarbonate, electrolytes, hemoglobin, albumin, C-reactive protein (CRP), and urine protein-to-creatinine ratio.

### Frailty assessment

Frailty was assessed at baseline, 12 months, and 24 months using the Fried frailty phenotype, operationalized according to the original criteria described in the Cardiovascular Health Study by Fried et al. [20].

The phenotype comprises five components: unintentional weight loss, exhaustion, weakness (grip strength), slowness (gait speed), and low physical activity. Each component was scored as present (1) or absent (0), yielding a total score from 0 to 5. Participants were categorized as robust (0 points), pre-frail (1–2 points), or frail (≥ 3 points).

All assessments were performed by a single trained investigator under standardized conditions and blinded to serum bicarbonate levels. The same operational definitions and cut-off values were applied at all time points.

Unintentional weight loss was defined as a loss of > 4.5 kg or > 5% of body weight over the preceding 12 months. Exhaustion was assessed using two items from the Center for Epidemiologic Studies Depression Scale (CES-D), and the criterion was met if either item was endorsed on ≥ 3 days per week.

Grip strength was measured with a calibrated hydraulic dynamometer (EH101, Camry Electronic Co., Ltd., Zhongshan, China) while participants were seated in a standardized position. Three measurements were obtained from the dominant hand, and the highest value was used for analysis.

Gait speed was assessed using a 4-meter walk test at a usual pace, and the mean of two trials was used for analysis.

Physical activity was assessed using the Minnesota Leisure Time Physical Activity Questionnaire (MLTPAQ), and weekly energy expenditure was calculated using standardized metabolic equivalents (METs) values.

The operational definitions and component-specific thresholds for frailty are presented in Table 1.

**Table 1:** Operational definitions and thresholds used for the fried frailty phenotype

Component	Measurement method	Cut-off definition
Weight loss	Self-report + medical record confirmation	> 4.5 kg or > 5% body weight (12 months)
Exhaustion	CES-D (2 items)	≥ 3 days/week (either item)
Weakness	Max grip strength (dominant hand, 3 trials)	Men: <29 kg (BMI ≤ 24), < 30 kg (BMI 24.1–28), < 32 kg (BMI > 28) Women: <17 kg (BMI ≤ 23), < 17.3 kg (BMI 23.1–26), < 18 kg (BMI 26.1–29), < 21 kg (BMI > 29)
Slowness	Mean 4-m walk time (2 trials)	Men: ≥7 s (≤ 173 cm), ≥ 6 s (> 173 cm) Women: ≥7 s (≤ 159 cm), ≥ 6 s (> 159 cm)
Low physical activity	MLTPAQ (kcal/week)	< 383 kcal/week (men); <270 kcal/week (women)

Functional tests were performed according to standardized geriatric assessment protocols. Component-specific thresholds were derived from the original Fried frailty phenotype definitions

*BMI* Body mass index, *CES-D* Center for epidemiologic studies depression scale, *MLTPAQ *Minnesota leisure time physical activity questionnaire, *kg *Kilograms, *cm *Centimeters; *m *Meters, *s *Seconds, *kcal* Kilocalories

### Outcomes

The primary outcome was longitudinal frailty status over 24 months, assessed at baseline, 12 months, and 24 months using the Fried phenotype and categorized as robust (0), pre-frail (1–2), or frail (≥ 3).

The key secondary outcome was frailty at 24 months, defined as frail (Fried score ≥ 3) versus non-frail (Fried score < 3). As a supportive analysis, the Fried frailty score (0–5) was analyzed as a quasi-continuous variable.

Exploratory outcomes included incident frailty among participants non-frail at baseline, transitions between frailty states during follow-up, and individual components of the Fried phenotype.

### Statistical analysis

Baseline characteristics were compared across acidosis persistence groups using ANOVA/Kruskal–Wallis or chi-square/Fisher’s exact tests, as appropriate. Standardized mean differences (SMDs) ≥ 0.25 were considered meaningful imbalances.

The primary outcome (longitudinal frailty category) was analyzed using mixed-effects ordinal logistic regression with patient-level random intercepts. Fixed effects included acidosis persistence category (ordinal: 0–3), time (0, 12, 24 months), and their interaction. Results are reported as proportional odds ratios (pORs) with 95% confidence intervals (CIs). The proportional odds assumption was verified using the Brant test. Three hierarchical models were fitted: (1) unadjusted; (2) adjusted for age, sex, time since transplantation, donor type, baseline eGFR, baseline hemoglobin, CCI, and baseline CRP; (3) Model 2 plus time-varying eGFR, representing the most conservative adjustment for kidney function.

The key secondary outcome (frailty at 24 months) was analyzed using logistic regression (unadjusted and adjusted for Model 2 covariates plus baseline Fried score), with tests for linear trend across ordered categories. Supportive analyses included Kruskal–Wallis tests and linear mixed-effects models for the Fried score.

Exploratory outcomes used logistic regression (incident frailty), chi-square tests (transitions, components), and Kruskal–Wallis (change scores); details in Supplement.

Additional sensitivity analyses addressed residual confounding, exposure definition, and temporal directionality. Propensity score–based inverse probability weighting (IPW) was performed with a multinomial ordinal model that included baseline eGFR, hemoglobin, CRP, CCI, time since transplantation, anti-HLA antibody status, and donor type. Stratified analyses by baseline eGFR tertile (< 46, 46–65, and > 65 mL/min/1.73 m²) were also conducted.

To assess the robustness of the exposure definition, bicarbonate was analyzed as a continuous variable using mean, nadir, and time-varying measures in mixed-effects models. To explore reverse causation, analyses were repeated after excluding participants who were frail at baseline (Fried score ≥ 3), and lagged models were constructed to relate prior acidosis exposure to subsequent frailty outcomes. Given the non-random allocation of alkali therapy, analyses were repeated after excluding treated patients and by including alkali supplementation as a covariate.

Missing data were handled under the missing-at-random assumption in mixed-effects models. All tests were two-sided; α = 0.05 without correction for multiple comparisons. All statistical analyses were performed in R software (R Foundation for Statistical Computing, Vienna, Austria) using RStudio version 2026.01.0 (Posit Software, PBC, Boston, MA, USA). Effect measures vary by analysis type: proportional odds ratios (pORs) are reported for ordinal mixed-effects models of the primary outcome; conventional odds ratios (ORs) for logistic regression analyses of frailty at 24 months and exploratory outcomes; and regression coefficients (β) for linear mixed-effects models of the Fried score.

## Results

### Study population

**Fig. 2:**
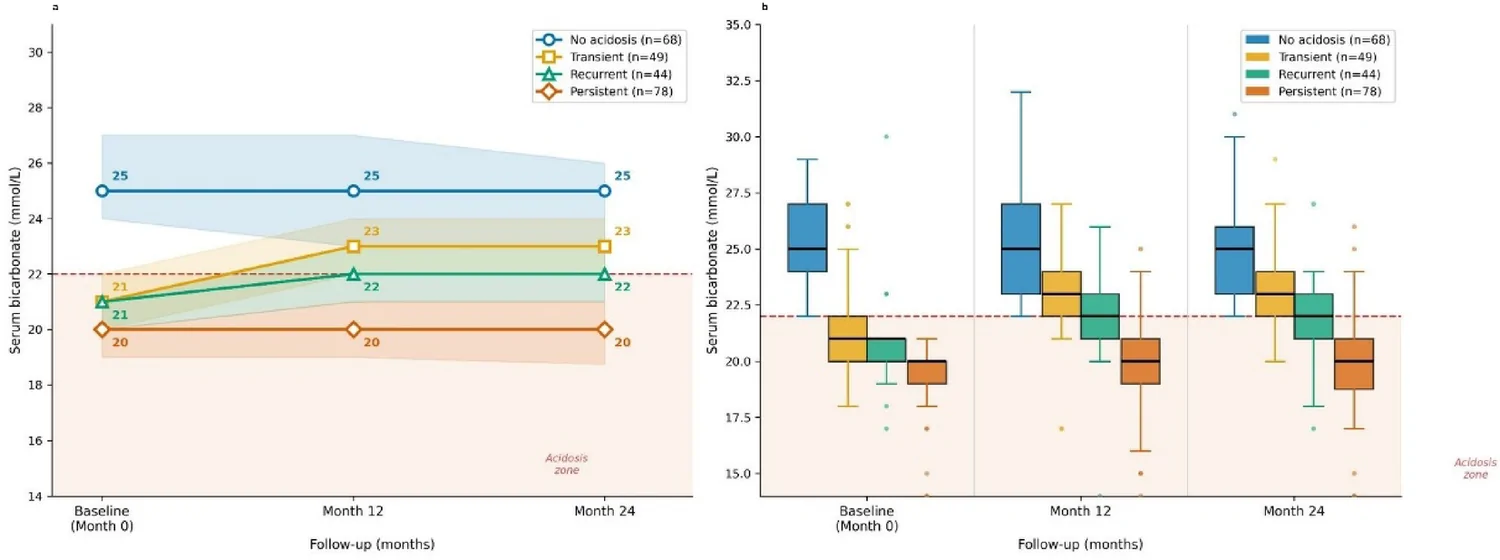
Longitudinal serum bicarbonate trajectories according to metabolic acidosis persistence group. **a** shows median serum bicarbonate levels with interquartile ranges across study visits (baseline, 12 months, and 24 months). **b** displays the distribution of bicarbonate values at each timepoint using boxplots. The dashed red line indicates the threshold defining metabolic acidosis (bicarbonate < 22 mmol/L)

Among the 239 kidney transplant recipients in the study, 68 (28.5%) had no acidosis (0 of 4 visits with bicarbonate < 22 mmol/L), 49 (20.5%) had transient acidosis (1 of 4 visits), 44 (18.4%) had recurrent acidosis (2 of 4 visits), and 78 (32.6%) had persistent acidosis (≥ 3 of 4 visits). Follow-up completion was 97.5% (233/239 participants); 6 patients were censored (1 death, 2 graft failures, and 3 loss to follow-up). Longitudinal serum bicarbonate trajectories across these groups are shown in Fig. 2.

### Baseline characteristics

Baseline characteristics across acidosis persistence categories are presented in Table 2.

**Table 2:** Baseline characteristics according to metabolic acidosis persistence group

Variable	Without acidosis (*n* = 68)	Transient (*n* = 49)	Recurrent (*n* = 44)	Persistent (*n* = 78)	Max SMD
*Demographic characteristics*					
Age, years — median [IQR]	47.0 [40.0–55.0]	44.0 [37.0–52.0]	46.0 [39.0–56.0]	48.0 [37.0–54.0]	0.19
Male sex — n (%)	43 (63.2)	27 (55.1)	27 (61.4)	46 (59.0)	0.14
BMI, kg/m² — median [IQR]	25.4 [22.0–28.2]	25.8 [23.3–27.7]	26.2 [22.0–28.2]	26.2 [22.5–28.8]	0.14
SBP, mmHg — median [IQR]	142.0 [134.0–150.0]	139.0 [130.0–148.0]	145.0 [135.0–150.0]	136.0 [128.0–148.0]	0.25
DBP, mmHg — median [IQR]	90.0 [80.0–98.0]	87.0 [78.0–92.0]	87.0 [80.0–98.0]	85.0 [75.0–93.0]	0.27
*Transplant characteristics*					
Time since transplant, months — median [IQR]	44.0 [17.0–85.0]	34.0 [11.0–81.0]	62.0 [26.0–113.0]	59.0 [24.0–117.0]	0.31
Deceased donor — n (%)	38 (55.9)	25 (51.0)	19 (43.2)	37 (47.4)	0.25
Donor age, years — median [IQR]	47.0 [42.0–52.0]	45.0 [41.0–55.0]	52.0 [44.0–56.0]	54.0 [44.0–58.0]	0.40
HLA mismatch total — median [IQR]	2.0 [2.0–3.0]	3.0 [2.0–3.0]	3.0 [2.0–3.0]	3.0 [2.0–3.0]	0.59
Delayed graft function — n (%)	0 (0.0)	3 (6.1)	2 (4.5)	7 (9.0)	0.46
Anti-HLA antibodies — n (%)	15 (22.1)	23 (46.9)	27 (61.4)	37 (47.4)	0.83
Induction: basiliximab — n (%)	65 (95.6)	46 (93.9)	44 (100.0)	68 (87.2)	0.31
Induction: thymoglobulin — n (%)	2 (2.9)	2 (4.1)	1 (2.3)	11 (14.1)	0.38
*Immunosuppressive regimen*					
Tacrolimus — n (%)	45 (66.2)	33 (67.3)	30 (68.2)	59 (75.6)	0.15
Cyclosporine — n (%)	22 (32.4)	15 (30.6)	13 (29.5)	19 (24.4)	0.14
Mycophenolate mofetil/MPA — n (%)	63 (92.6)	49 (100.0)	43 (97.7)	75 (96.2)	0.40
Prednisone — n (%)	60 (88.2)	44 (89.8)	37 (84.1)	68 (87.2)	0.09
*Alkali therapy*					
Citrate/NaHCO₃ treatment — n (%)	0 (0.0)	27 (55.1)	29 (65.9)	25 (32.1)	2.00^a^
*Comorbidities*					
CCI = 0 — n (%)	47 (69.1)	35 (71.4)	22 (50.0)	42 (53.8)	0.35
CCI = 1 — n (%)	11 (16.2)	6 (12.2)	5 (11.4)	11 (14.1)	0.19
CCI ≥ 2 — n (%)	10 (14.7)	8 (16.3)	17 (38.6)	25 (32.1)	0.55
Hypertension — n (%)	53 (77.9)	40 (81.6)	38 (86.4)	66 (84.6)	0.22
Diabetes mellitus type 2 — n (%)	6 (8.8)	5 (10.2)	11 (25.0)	20 (25.6)	0.45
Atrial fibrillation — n (%)	2 (2.9)	0 (0.0)	4 (9.1)	5 (6.4)	0.29
Coronary heart disease — n (%)	6 (8.8)	4 (8.2)	11 (25.0)	14 (17.9)	0.50
Heart failure — n (%)	3 (4.4)	1 (2.0)	7 (15.9)	3 (3.8)	0.43
Anemia — n (%)	13 (19.1)	16 (32.7)	24 (54.5)	55 (70.5)	1.18
Dyslipidemia — n (%)	45 (66.2)	35 (71.4)	27 (61.4)	49 (62.8)	0.13
Obesity — n (%)	9 (13.2)	7 (14.3)	7 (15.9)	13 (16.7)	0.09
*Laboratory parameters at baseline*					
eGFR, mL/min/1.73 m² — median [IQR]	68.0 [55.0–81.0]	56.0 [47.0–74.0]	45.0 [37.0–57.0]	43.0 [28.0–61.0]	1.17
Creatinine, mg/dL — median [IQR]	1.2 [1.0–1.4]	1.3 [1.1–1.6]	1.6 [1.4–2.0]	1.7 [1.4–2.2]	1.05
Bicarbonate, mmol/L — median [IQR]	25.5 [24.0–27.0]	21.0 [20.0–22.0]	21.0 [20.0–21.0]	20.0 [19.0–20.0]	3.39
Hemoglobin, g/dL — median [IQR]	13.6 [12.2–14.6]	12.4 [11.4–13.4]	11.7 [10.2–13.1]	11.0 [10.0–12.4]	0.84
Potassium, mEq/L — median [IQR]	4.0 [4.0–5.0]	4.0 [4.0–5.0]	4.0 [4.0–5.0]	4.0 [4.0–5.0]	0.22
C-reactive protein, mg/L — median [IQR]	3.1 [2.1–4.5]	4.1 [2.3–5.8]	6.1 [4.5–7.8]	6.1 [3.1–8.6]	0.89
UPCR, g/g — median [IQR]	0.00 [0.00–0.04]	0.02 [0.00–0.10]	0.04 [0.00–0.85]	0.17 [0.00–1.07]	0.62
Log₂(UPCR) — median [IQR]	−6.64 [− 6.64–−4.38]	−6.64 [− 6.64–−3.36]	−6.64 [− 6.64–−0.18]	−2.00 [− 6.64–0.15]	0.83
*Frailty assessment—Fried phenotype*					
Fried frailty score (0–5) — median [IQR]	1.0 [0.0–1.0]	1.0 [0.0–1.0]	1.0 [0.0–2.0]	1.0 [0.0–2.0]	0.54
Robust (score 0) — n (%)	29 (42.6)	21 (42.9)	13 (29.5)	24 (30.8)	0.54
Pre-frail (score 1–2) — n (%)	36 (52.9)	24 (49.0)	24 (54.5)	37 (47.4)	0.21
Frail (score ≥ 3) — n (%)	3 (4.4)	4 (8.2)	8 (18.2)	17 (21.8)	0.50
Handgrip strength, kgf — median [IQR]	26.0 [22.0–31.0]	26.0 [22.5–29.5]	26.0 [22.0–29.0]	24.0 [22.0–27.0]	0.32
Gait speed, m/s — median [IQR]	1.1 [1.0–1.2]	1.2 [1.1–1.4]	1.1 [1.0–1.2]	1.1 [0.9–1.2]	0.43
Low physical activity — n (%)	11 (16.2)	9 (18.4)	14 (31.8)	21 (26.9)	0.34
Weight loss (> 4.5 kg or > 5%) — n (%)	0 (0.0)	1 (2.0)	4 (9.1)	10 (12.8)	0.55
Exhaustion — n (%)	10 (14.7)	7 (14.3)	12 (27.3)	28 (35.9)	0.47

Data are presented as median [IQR] for continuous variables and n (%) for categorical variables. Standardized mean differences (SMD) ≥ 0.25 were considered indicative of meaningful imbalance. Acidosis persistence groups were defined as follows: no acidosis = 0/4 visits with bicarbonate < 22 mmol/L; transient = 1/4 visits; recurrent = 2/4 visits; persistent = ≥ 3/4 visits.

^a^SMD for citrate/NaHCO₃ treatment is large by design.

*BMI *Body mass index, *SBP* Systolic blood pressure, *DBP* Diastolic blood pressure, *HLA* Human leukocyte antigen, *MMF* Mycophenolate mofetil, *MPA *Mycophenolic acid, *eGFR* Estimated glomerular filtration rate (CKD-EPI 2021), *UPCR* Urine protein-to-creatinine ratio, *CRP* C-reactive protein, *CCI* Charlson comorbidity index, *SMD* Standardized mean difference, *IQR* Interquartile range.

Patients with more persistent acidosis had progressively lower kidney function at baseline. The median eGFR decreased from 68 mL/min/1.73 m² [55–81] in patients without acidosis to 43 mL/min/1.73 m² [28–61] in those with persistent acidosis. Greater persistence of acidosis was also associated with a higher inflammatory burden, with the median C-reactive protein rising from 3.1 mg/L [2.1–4.5] to 6.1 mg/L [3.1–8.6] across groups.

The prevalence of several comorbid conditions was also higher among patients with recurrent or persistent acidosis, particularly anemia (19.1% vs. 70.5%) and diabetes mellitus (8.8% vs. 25.6%). Vascular stiffness, assessed by pulse wave velocity, increased across acidosis persistence categories (8.1 vs. 9.9 m/s).

### Primary outcome: longitudinal frailty category trajectory

Frailty category distributions differed significantly across acidosis persistence groups at all timepoints (baseline *p* = 0.050, 12 months *p* = 0.004, 24 months *p* = 0.021; Table 3). Across follow-up, patients with greater acidosis persistence exhibited progressively worse frailty profiles.

**Table 3:** Frailty category distribution according to metabolic acidosis persistence (n, **%)**

Timepoint	Frailty Category	Without acidosis, *n* (%)	Transient acidosis, *n* (%)	Recurrent acidosis, *n* (%)	Persistent acidosis, *n* (%)	*p* (Chi-square)
Baseline	Robust	29 (42.6)	21 (42.9)	13 (29.5)	24 (30.8)	0.050
	Pre-frail	36 (52.9)	24 (49.0)	24 (54.5)	37 (47.4)	
	Frail	3 (4.4)	4 (8.2)	7 (15.9)	17 (21.8)	
12 months	Robust	25 (36.8)	22 (44.9)	15 (34.9)	19 (24.4)	0.004
	Pre-frail	39 (57.4)	22 (44.9)	19 (44.2)	36 (46.2)	
	Frail	4 (5.9)	5 (10.2)	9 (20.9)	23 (29.5)	
24 months	Robust	27 (39.7)	22 (44.9)	10 (23.3)	20 (27.4)	0.021
	Pre-frail	36 (52.9)	20 (40.8)	24 (55.8)	33 (45.2)	
	Frail	5 (7.4)	7 (14.3)	9 (20.9)	20 (27.4)	

Sample sizes: Without acidosis (*n* = 68), Transient acidosis (*n* = 49), Recurrent acidosis (*n* = 44), Persistent acidosis (*n* = 78). Values are expressed as n (%). Percentages at 24 months were calculated among participants with available follow-up. Chi-square tests compare frailty distribution across acidosis persistence groups at each timepoint

At 24 months, 39.7% of patients without acidosis remained robust, compared with 27.4% of those with persistent acidosis, whereas the prevalence of frailty increased from 7.4% to 27.4%.

In unadjusted mixed-effects ordinal logistic regression (Table 4, Model 1), longer acidosis persistence was associated with higher odds of a worse frailty category (pOR per category 1.91, 95% CI 1.42–2.56; *p* < 0.001). Compared with patients without acidosis, those with transient acidosis (pOR 1.91, 95% CI 1.24–2.94), recurrent acidosis (pOR 3.65, 95% CI 2.25–5.92), and persistent acidosis (pOR 5.67, 95% CI 3.63–8.86) had higher odds of a worse frailty category.

**Table 4:** Mixed-effects ordinal logistic regression for frailty category according to metabolic acidosis persistence over 24 months

Variable	Model 1 Unadjusted pOR (95% CI)	*p* value	Model 2 Adjusted pOR (95% CI)	*p* value	Model 3 Adjusted pOR (95% CI)	*p* value
Acidosis persistence (per category)	1.91 (1.42–2.56)	< 0.001	1.52 (1.12–2.06)	0.008	1.42 (1.04–1.95)	0.029
Transient vs. Without	1.91 (1.24–2.94)	0.003	1.61 (1.02–2.54)	0.041	1.54 (0.97–2.45)	0.067
Recurrent vs. Without	3.65 (2.25–5.92)	< 0.001	2.71 (1.59–4.61)	< 0.001	2.41 (1.39–4.17)	0.002
Persistent vs. Without	5.67 (3.63–8.86)	< 0.001	3.97 (2.12–7.42)	< 0.001	3.29 (1.66–6.51)	0.002
Time (per 12 months)	1.18 (0.97–1.44)	0.102	1.15 (0.94–1.40)	0.169	1.08 (0.88–1.32)	0.461
Time × Acidosis	—	0.756	—	0.815	—	0.883
Concurrent eGFR (per 10 mL/min decrease)	—	—	—	—	1.23 (1.09–1.39)	0.001

Model 1 unadjusted.

Model 2 adjusted for age, sex, time since transplantation, donor type, baseline eGFR, hemoglobin, Charlson Comorbidity Index, and C-reactive protein.

Model 3 Model 2 with additional adjustment for concurrent (time-varying) eGFR.

*pOR* Proportional odds ratio, *CI* Confidence interval, *eGFR* Estimated glomerular filtration rate.

After adjusting for baseline clinical and transplant characteristics (Table 4, Model 2), persistent acidosis remained independently associated with a worse frailty category (pOR 3.97, 95% CI 2.12–7.42; *p* < 0.001). In the adjusted models, lower baseline eGFR (per 10 mL/min decrease: pOR 1.18, 95% CI 1.05–1.32; *p* = 0.005) and lower hemoglobin (per 1 g/dL decrease: pOR 1.12, 95% CI 1.02–1.23; *p* = 0.018) were also associated with worse frailty status.

In the most conservative model, which included time-varying kidney function (Table 4, Model 3), persistent acidosis remained significantly associated with a worse frailty category (pOR 3.29, 95% CI 1.66–6.51; *p* = 0.002). Concurrent eGFR was independently associated with frailty trajectory (pOR per 10 mL/min decrease 1.23, 95% CI 1.09–1.39; *p* = 0.001).

The time × acidosis interaction was not statistically significant, indicating that the relative differences in frailty category across acidosis persistence groups remained broadly stable over time.

**Fig. 3:**
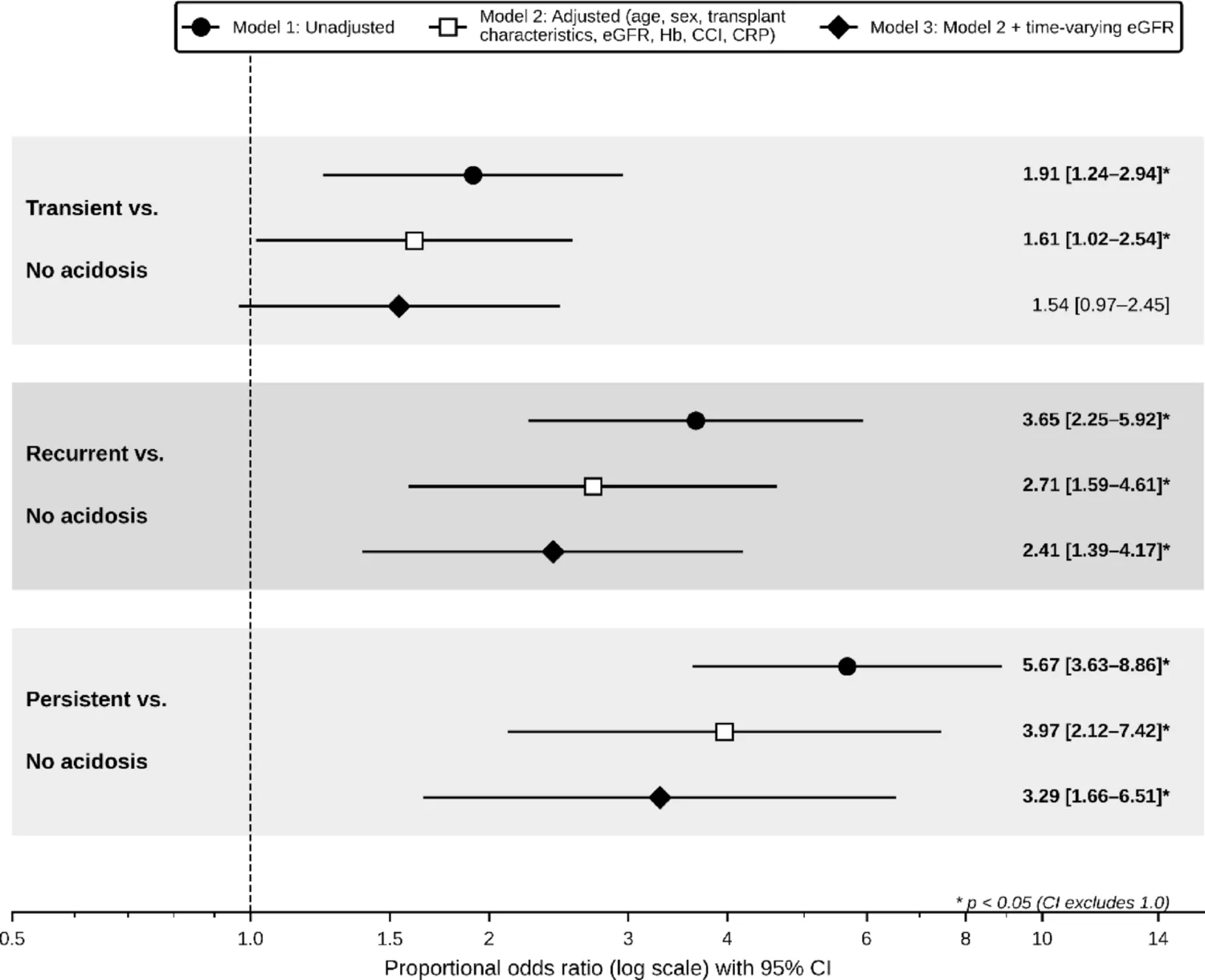
Association between metabolic acidosis persistence and longitudinal frailty category Forest plot showing proportional odds ratios for worse frailty category according to acidosis persistence across three hierarchical mixed-effects models. Model 1 is unadjusted; Model 2 is adjusted for age, sex, transplant characteristics, baseline eGFR, hemoglobin, Charlson Comorbidity Index, and C-reactive protein; Model 3 additionally includes time-varying eGFR. Points represent odds ratios and horizontal lines indicate 95% confidence intervals

Effect estimates across the three hierarchical models are summarized in Fig. 3.

### The key secondary outcome: frailty at 24 months

Frailty prevalence at 24 months increased progressively across acidosis persistence categories, from 7.4% (5/68) in patients without acidosis to 14.3% (7/49) with transient acidosis, 20.9% (9/44) with recurrent acidosis, and 27.4% (21/78) with persistent acidosis (χ² *p* = 0.017; linear trend *p* = 0.004) (Table 5).

**Table 5:** Association between metabolic acidosis persistence and frailty at 24 months

Acidosis persistence group	Frail, *n* (%)	Unadjusted OR (95% CI)	*p* value	Adjusted OR^a^ (95% CI)	*p* value
Without acidosis	5 (7.4)	Reference	—	Reference	—
Transient acidosis	7 (14.3)	2.10 (0.62–7.06)	0.230	1.84 (0.51–6.62)	0.351
Recurrent acidosis	9 (20.9)	3.34 (1.04–10.75)	0.044	2.72 (0.79–9.36)	0.113
Persistent acidosis	21 (27.4)	4.75 (1.67–13.53)	0.002	3.28 (1.08–9.95)	0.036

Values are n (%) unless otherwise indicated. Odds ratios (ORs) and 95% confidence intervals (CIs) were estimated using logistic regression with frailty at 24 months (Fried score ≥ 3) as the outcome and the “without acidosis” group as reference.

^a^Adjusted models included age, sex, transplant characteristics, baseline eGFR, hemoglobin, Charlson Comorbidity Index, and baseline Fried score.

In unadjusted logistic regression, persistent acidosis was associated with substantially higher odds of frailty at 24 months than in the absence of acidosis (OR 4.75, 95% CI 1.67–13.53; *p* = 0.002).

After adjusting for age, sex, transplant characteristics, baseline eGFR, hemoglobin, CCI, and baseline Fried score, persistent acidosis remained independently associated with frailty at 24 months (adjusted OR 3.28, 95% CI 1.08–9.95; *p* = 0.036).

A dose–response relationship across ordered acidosis persistence categories was observed in both the unadjusted (*p* = 0.003) and the adjusted models (*p* = 0.030).

As a supportive analysis, the Fried frailty score (0–5) was treated as a quasi-continuous variable in linear mixed-effects models. Longer acidosis persistence was associated with a higher frailty score (β 0.31 per category increase, 95% CI 0.18–0.44; *p* < 0.001).

Time was not significantly associated with the frailty score (β 0.08 per 12-month increase, 95% CI − 0.02 to 0.18; *p* = 0.112), and the time × acidosis interaction was not statistically significant (β − 0.02, 95% CI − 0.08 to 0.04; *p* = 0.459) (Supplementary Table S1).

### Exploratory outcomes

#### Incident frailty

These analyses should be considered exploratory given the small number of incident events and the resulting limited statistical power. Among the 205 participants who were non-frail at baseline, 14 (6.8%) developed frailty during the 24-month follow-up. The incidence of frailty increased across acidosis persistence categories, with 2 of 65 (3.1%) patients without acidosis, 3 of 45 (6.7%) with transient acidosis, 3 of 37 (8.1%) with recurrent acidosis, and 6 of 58 (10.3%) with persistent acidosis (Supplementary Table S2).

#### Frailty transitions

Transitions between frailty states from baseline to 24 months also differed across acidosis persistence groups. Worsening transitions occurred in 8 of 68 (11.8%) patients without acidosis, compared with 18 of 73 (24.7%) among those with persistent acidosis.

**Fig. 4:**
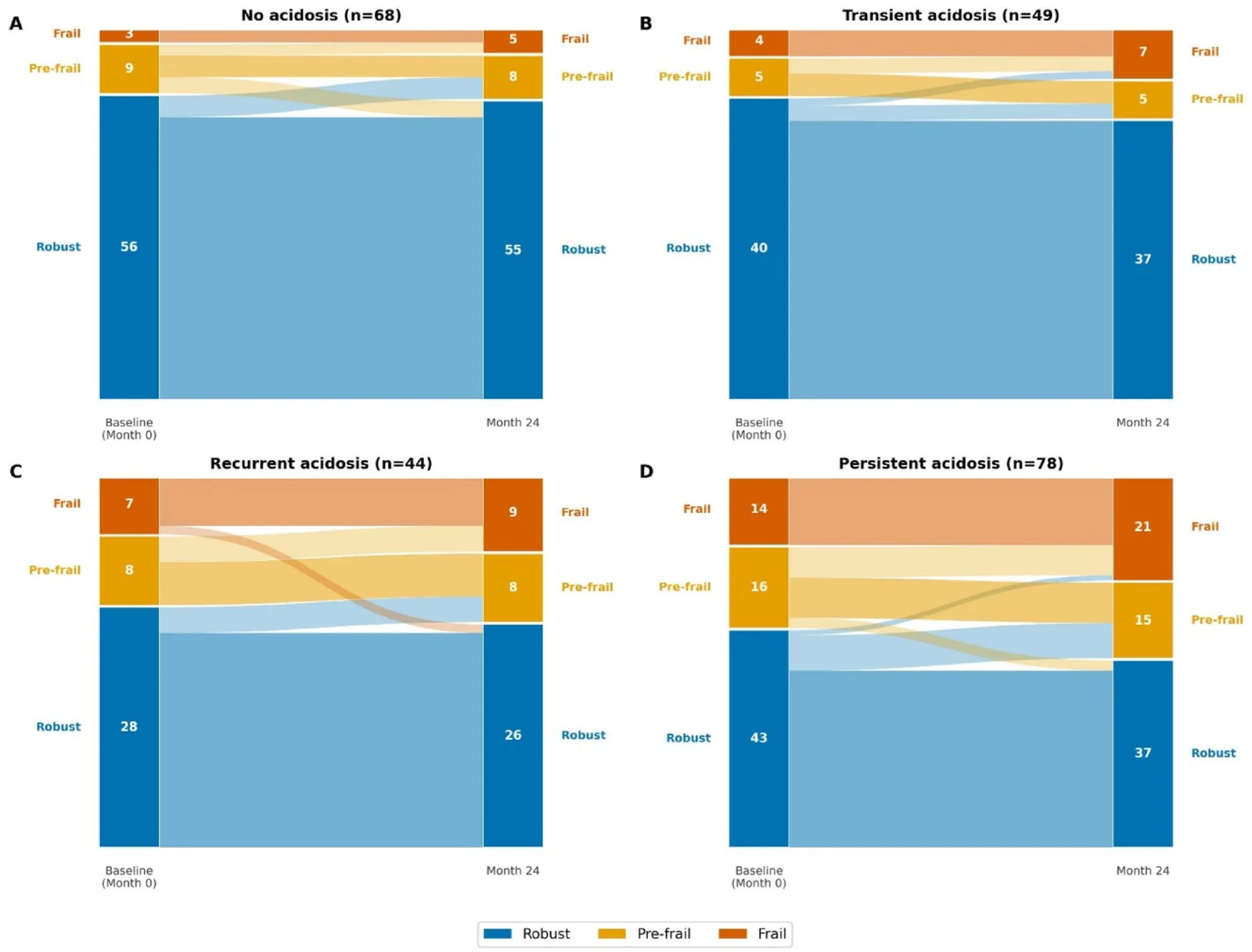
Frailty state transitions from baseline to 24 months according to metabolic acidosis persistence Alluvial diagrams illustrate transitions between frailty states (robust, pre-frail, and frail) over the 24-month follow-up period within each acidosis persistence group. **A** Patients without metabolic acidosis (0/4 visits with bicarbonate < 22 mmol/L; *n* = 68). **B **Patients with transient acidosis (1/4 visits; *n* = 49). **C **Patients with recurrent acidosis (2/4 visits; *n* = 44). **D **Patients with persistent acidosis (≥ 3/4 visits; *n* = 78)

In contrast, improvement in frailty status was observed in 6 of 68 (8.8%) patients without acidosis and in 5 of 73 (6.8%) with persistent acidosis (Supplementary Table S3). Frailty state transitions by acidosis persistence group are illustrated in Fig. 4.

#### Individual frailty components

The prevalence of several individual frailty components varied across acidosis persistence categories at both baseline and 24 months, particularly exhaustion, weakness, slowness, and weight loss. In contrast, low physical activity did not differ significantly between groups (Supplementary Table S4).

### Sensitivity analyses

Given the differential distribution of alkali supplementation across acidosis persistence groups (0.0%, 57.1%, 65.9%, and 32.1% in the no acidosis, transient, recurrent, and persistent groups, respectively), two pre-specified sensitivity analyses were conducted to assess whether the association between persistent acidosis and frailty could be attributable to differential treatment exposure.

First, the analysis was restricted to patients who did not receive alkali therapy (no acidosis: *n* = 68; persistent acidosis: *n* = 53). Among alkali-untreated patients, frailty at 24 months occurred in 5 of 68 (7.4%) without acidosis and in 12 of 53 (22.6%) with persistent acidosis. The association remained statistically significant (OR 3.98, 95% CI 1.07–14.81; *p* = 0.016). Within the persistent acidosis group, frailty prevalence was numerically higher among alkali-treated patients (8/25, 32.0%) than among untreated patients (12/53, 22.6%), but the difference was not statistically significant (*p* = 0.401).

Second, alkali treatment was included as a covariate in the fully adjusted logistic regression model (Persistent vs. No acidosis, *n* = 146 complete cases). Alkali treatment was not independently associated with frailty at 24 months (OR 0.89, 95% CI 0.15–5.24; *p* = 0.896). These results are presented in Supplementary Tables S5-S6. These analyses suggest that the primary association was not entirely explained by measured differences in alkali treatment exposure; however, because treatment allocation was non-random, they do not permit inference regarding the effect of alkali supplementation itself.

In propensity score–weighted analyses, the association between persistent acidosis and frailty at 24 months remained significant (IPW-weighted OR per category 1.43, 95% CI 1.02–2.00, *p* = 0.037; Supplementary Table S7). These eGFR-stratified analyses should be considered exploratory given the small number of frailty events. Models in the higher eGFR strata did not converge because of insufficient events. The apparent concentration of effect in the lowest eGFR tertile (< 46 mL/min/1.73 m²; adjusted OR 4.67, *p* = 0.086 for trend; Supplementary Table S8) should be interpreted cautiously and considered hypothesis-generating. In sensitivity analyses using continuous bicarbonate measures, each 1 mmol/L decrease in mean bicarbonate was associated with a 59% higher adjusted odds of frailty at 24 months (OR 1.59, 95% CI 1.10–2.29, *p* = 0.013; Supplementary Table S9). Consistent results were observed with the nadir bicarbonate value (OR 1.49, 95% CI 1.07–2.07, *p* = 0.018; Supplementary Table S10) and in time-varying mixed-effects models of the Fried score (β + 0.025 per mmol/L decrease, *p* = 0.004; Supplementary Table S11).

In analyses restricted to non-frail participants at baseline (*n* = 208), incident frailty at 24 months increased progressively across acidosis persistence categories (3.2% to 12.7%), showing a significant dose-response trend (Spearman *r* = 0.140, *p* = 0.045; Supplementary Table S12). In lagged exposure analyses, cumulative acidosis at visits 1–2 significantly predicted a worse frailty category at 12 months (pOR 2.09, 95% CI 1.29–3.39, *p* = 0.003; Supplementary Table S13), providing temporal support for the proposed direction of effect.

## Discussion

In this prospective cohort of kidney transplant recipients, persistent metabolic acidosis was associated with a higher likelihood of worsening frailty category over 24 months. A graded increase in frailty risk was observed across ordered categories of acidosis persistence, and this association remained significant after adjustment for baseline characteristics, comorbidity burden, and concurrent kidney function. The persistence of the association in propensity score–weighted analyses (IPW-OR 1.43, 95% CI 1.02–2.00) suggests that this relationship is not fully explained by measured differences in disease severity, although residual confounding cannot be excluded. These eGFR-stratified analyses are exploratory and limited by small numbers of events; models in higher eGFR strata did not converge due to insufficient frailty events. The apparent concentration of effect in the lowest eGFR tertile (< 46 mL/min/1.73 m²; adjusted OR 4.67, *p* = 0.086 for trend; Supplementary Table S8) should be interpreted with caution: the absence of a detectable association in higher strata most likely reflects limited statistical power rather than a true null effect, and no conclusion regarding eGFR as an effect modifier can be drawn from these data. To our knowledge, this is the first prospective study to examine the longitudinal relationship between acidosis persistence—defined across repeated assessments—and frailty trajectory in kidney transplant recipients.

Frailty after kidney transplantation follows a dynamic trajectory, with early improvement followed by later decline, as shown in prior longitudinal studies. In a large prospective cohort, Chu et al. found that the likelihood of frailty decreased during the first 2.5 years post-transplant but increased thereafter, with deterioration in strength and physical activity [21]. In our cohort, frailty prevalence at 24 months ranged from 7.4% in patients without acidosis to 27.4% in those with persistent acidosis, consistent with published Western transplant cohorts. While longitudinal frailty data are predominantly derived from North American populations, evidence from Eastern European cohorts remains limited, underscoring the relevance of our findings [5, 6].

Metabolic acidosis affects 12–58% of kidney transplant recipients and can occur even with relatively preserved eGFR, likely due to transplant-specific mechanisms such as calcineurin inhibitor–related tubular dysfunction and reduced nephron mass. Tacrolimus and other calcineurin inhibitors impair acid–base transport in renal tubules, and dose- and duration-dependent toxicity can lead to renal tubular acidosis [22, 23]. Prior studies have primarily focused on graft survival and mortality, with limited data on functional decline. In large cohorts of kidney transplant recipients, lower serum bicarbonate levels have consistently been associated with increased risk of graft loss (HR 1.74, 95% CI 1.26–2.42) and death-censored graft failure [24]. Our classification of acidosis, based on persistence across repeated assessments, was designed to capture cumulative exposure, which may be more relevant to functional outcomes than a single measurement.

The attenuation of effect estimates after including time-varying eGFR (Model 3: pOR 3.29, 95% CI 1.66–6.51) suggests that kidney function accounts for part of the observed association; formal mediation analysis indicated that this proportion is modest (Supplementary Table S14), though causal interpretation is limited by the observational design.

Several biological mechanisms may explain the association between metabolic acidosis and frailty. Acidosis promotes skeletal muscle proteolysis by activating the ubiquitin–proteasome pathway, contributing to muscle mass loss and reduced physical performance [25]. It may also impair mitochondrial function and reduce ATP production, leading to fatigue and decreased physical endurance. At the systemic level, metabolic acidosis is linked to a pro-inflammatory and hormonal milieu that may further promote catabolism and functional decline [17]. Consistent with these mechanisms, interventional studies in non-dialysis CKD populations have shown improvements in muscle mass and physical function after correcting metabolic acidosis [26]. Whether similar effects occur in kidney transplant recipients remains to be established.

Interpretation of alkali supplementation is limited by confounding by indication. Treatment rates varied across groups, with lower use among patients with persistent acidosis despite a greater cumulative acid burden, likely reflecting real-world clinical decision-making.

In sensitivity analyses, the association between persistent acidosis and frailty remained after restriction to alkali-untreated patients (OR 3.98, 95% CI 1.07–14.81; *p* = 0.016) and was not materially altered by adjustment for alkali therapy. Alkali therapy itself was not independently associated with frailty outcome (OR 0.89, 95% CI 0.15–5.24; *p* = 0.896). Given non-random treatment allocation, no causal inference regarding alkali supplementation can be drawn.

The consistency of findings across multiple sensitivity analyses—including propensity score weighting, alternative bicarbonate definitions, and restriction to non-frail participants—supports the robustness of the observed association. Notably, the association was attenuated when acidosis was defined by a single baseline measurement, suggesting that persistent rather than isolated disturbances are more relevant to functional decline.

Reverse causation cannot be excluded, as frailty may influence acid-base status through mechanisms such as malnutrition or inflammation. However, analyses excluding baseline frail participants and lagged models yielded directionally consistent but attenuated results, supporting—though not proving—a temporal relationship.

These findings have potential clinical implications. Serum bicarbonate is routinely measured after transplantation, and longitudinal patterns may offer additional prognostic information. If confirmed in interventional studies, correcting persistent acidosis may be a strategy to mitigate functional decline.

### Limitations

Several limitations warrant consideration. First, as this was an observational study, causal inference is not possible. Although propensity score–weighted analyses yielded directionally consistent results, residual confounding by unmeasured determinants of disease severity—including dietary acid load, pre-transplant frailty trajectory, dialysis vintage, and tubular dysfunction not captured by eGFR—cannot be excluded.

Second, this was a single-center study conducted in Eastern Romania. While it provides valuable data from an underrepresented population, generalizability to other settings may be limited. Third, classifying acidosis persistence by the number of visits with bicarbonate < 22 mmol/L does not capture intra-episode severity or temporal patterns. However, consistent results across analyses using mean, nadir, and time-varying bicarbonate support the robustness of this approach. Fourth, the number of incident frailty events was limited (*n* = 14), resulting in wide confidence intervals and non-significant adjusted estimates in the incident frailty and eGFR-stratified analyses. These analyses are therefore exploratory and should not be interpreted as definitive evidence.

The bidirectional relationship between frailty and metabolic acidosis cannot be excluded, as frailty-associated malnutrition or inflammation may influence acid–base status. Although sensitivity analyses excluding baseline frail participants and lagged models yielded directionally consistent results, temporal relationships cannot be definitively established without interventional data. Finally, analyses of alkali supplementation are subject to confounding by indication, as treatment was not randomly assigned. The absence of a significant association between alkali therapy and frailty should not be interpreted as evidence of a lack of efficacy, and randomized trials are required to determine its impact on functional outcomes.

## Conclusion

Persistent metabolic acidosis was associated with worsening frailty trajectories and higher frailty prevalence at 24 months among kidney transplant recipients, with a dose–response relationship across acidosis persistence categories. These findings suggest that persistent low bicarbonate may be a clinically relevant marker of functional decline, although causality cannot be established. Randomized trials are needed to determine whether correcting metabolic acidosis can reduce frailty risk or mitigate functional decline after kidney transplantation.

## Supplementary Information

Below is the link to the electronic supplementary material.

Supplementary Material 1
