# Supplementary material for: Persistent metabolic acidosis and frailty trajectories after kidney transplantation: a prospective cohort study
Source: Clin Exp Nephrol. 2026 Jul 27;30(11):1628–39. doi: 10.1007/s10157-026-02920-y (PMC13668929; doi:10.1007/s10157-026-02920-y)
Supplement: Supplementary file 1 [file 10157_2026_2920_MOESM1_ESM.docx]

**Supplementary Material**

**Manuscript title**: Persistent Metabolic Acidosis and Frailty Trajectories After Kidney Transplantation: A Prospective Cohort Study

**Authors:**

Lucian Siriteanu^1,2^, MD

Andreea Covic^1,2^, MD

Călin Namolovan^1,2^, MD

Simona Mihaela Hogaș^1,2^, MD, PhD

Luminița Voroneanu^1,2^, MD, PhD

Adrian Covic^1,2^, MD, PhD, FRCP (London), FRCP (Edin.), FERA, FESC

**Affiliations:**

^1^ Grigore T. Popa University of Medicine and Pharmacy

^2^ Dr. C. I. Parhon Hospital

**Correspondence to:**

Lucian Siriteanu, MD

Email: siriteanulucian@gmail.com

Dr. C. I. Parhon Hospital

Carol I Boulevard No. 50
Iași 700503
Romania

**Supplementary Table S1.** Linear Mixed-Effects Model for Fried Frailty Score

| **Variable** | **β (95% CI)** | **p value** |
| --- | --- | --- |
| Acidosis persistence (per category) | 0.31 (0.18–0.44) | <0.001 |
| Time (per 12 months) | 0.08 (−0.02–0.18) | 0.112 |
| Time × Acidosis persistence | −0.02 (−0.08–0.04) | 0.459 |
| **Abbreviations:** β, regression coefficient; CI, confidence interval.  Estimates are derived from a linear mixed-effects model treating Fried frailty score (0–5) as a quasi-continuous outcome. The model included fixed effects for acidosis persistence category, time, and their interaction, with patient-level random intercepts.  The analysis included 239 participants contributing 699 repeated measurements. | | |

**Supplementary Table S2.** Incident Frailty at 24 Months Among Participants Non-Frail at Baseline (n=205)

| **Acidosis persistence** | **Events / At risk** | **Incidence (%)** | **Unadjusted OR (95% CI)** | **p value** | **Adjusted OR^a^ (95% CI)** | **p value** |
| --- | --- | --- | --- | --- | --- | --- |
| Without acidosis | 2 / 65 | 3.1 | Reference | — | Reference | — |
| Transient acidosis | 3 / 45 | 6.7 | 2.25 (0.36–14.04) | 0.398 | 1.94 (0.29–13.03) | 0.489 |
| Recurrent acidosis | 3 / 37 | 8.1 | 2.86 (0.46–18.00) | 0.345 | 2.27 (0.33–15.67) | 0.403 |
| Persistent acidosis | 6 / 58 | 10.3 | 3.57 (0.69–18.41) | 0.127 | 2.94 (0.54–16.02) | 0.216 |
| Per-category trend (unadjusted): OR 1.82 (95% CI 1.01–3.29), p = 0.048.  Per-category trend (adjusted): OR 1.71 (95% CI 0.92–3.18), p = 0.089.  ^a^Adjusted for age, sex, baseline estimated glomerular filtration rate (eGFR), hemoglobin, and Charlson Comorbidity Index.  Incident frailty was defined as development of Fried score ≥3 at 24 months among participants non-frail at baseline (Fried score <3). Odds ratios (ORs) with 95% confidence intervals (CIs) were estimated using logistic regression models with the “without acidosis” group as reference. Linear trend across ordered acidosis persistence categories was assessed by modeling acidosis persistence as an ordinal variable.  Baseline status: robust 169 (82.4%), pre-frail 36 (17.6%). | | | | | | |

**Supplementary Table S3.** Frailty State Transitions From Baseline to 24 Months

| **Transition outcome** | **Without acidosis (n = 68)** | **Persistent acidosis (n = 73)** | **p value** |
| --- | --- | --- | --- |
| **Overall transitions** |  |  |  |
| Worsening (to worse frailty category) | 8 (11.8%) | 18 (24.7%) | 0.043 |
| Improvement (to better frailty category) | 6 (8.8%) | 5 (6.8%) | 0.777 |
| **Among participants robust at baseline** |  |  |  |
| Remained robust | 25 (86.2%) | 15 (62.5%) | 0.024 |
| Became frail | 1 (3.4%) | 3 (12.5%) | 0.092 |
| Frailty states were defined as robust (Fried score=0), pre-frail (Fried score=1–2), and frail (Fried score≥3). Transition outcomes were calculated from baseline to 24 months. Worsening was defined as movement to a worse frailty state and improvement as movement to a better frailty state. Group differences were assessed using chi-square tests.  Transition analyses were restricted to the extreme exposure groups (no acidosis vs persistent acidosis) to maximize contrast and avoid sparse transition cells in intermediate groups. | | | |

**Supplementary Table S4.** Prevalence of Individual Fried Frailty Components According to Acidosis Persistence and Timepoint

| **Component** | **Timepoint** | **Without** | **Transient** | **Recurrent** | **Persistent** | **p value** |
| --- | --- | --- | --- | --- | --- | --- |
| **Weakness** | Baseline | 20.6% | 26.5% | 30.2% | 41.0% | 0.028 |
|  | 12 months | 22.1% | 28.6% | 34.9% | 44.9% | 0.012 |
|  | 24 months | 23.5% | 30.6% | 37.2% | 47.9% | 0.008 |
| **Slowness** | Baseline | 14.7% | 18.4% | 23.3% | 32.1% | 0.048 |
|  | 12 months | 16.2% | 22.4% | 27.9% | 35.9% | 0.032 |
|  | 24 months | 17.6% | 24.5% | 30.2% | 38.4% | 0.021 |
| **Exhaustion** | Baseline | 14.7% | 24.5% | 32.6% | 43.6% | <0.001 |
|  | 12 months | 19.1% | 32.7% | 39.5% | 50.0% | <0.001 |
|  | 24 months | 20.6% | 34.7% | 41.9% | 52.1% | <0.001 |
| **Low physical activity** | Baseline | 17.6% | 20.4% | 27.9% | 30.8% | 0.174 |
|  | 12 months | 19.1% | 22.4% | 30.2% | 33.3% | 0.145 |
|  | 24 months | 20.6% | 24.5% | 32.6% | 35.6% | 0.128 |
| **Weight loss** | Baseline | 0.0% | 2.0% | 4.7% | 10.3% | 0.012 |
|  | 12 months | 4.4% | 8.2% | 11.6% | 20.5% | 0.006 |
|  | 24 months | 5.9% | 10.2% | 14.0% | 23.3% | 0.004 |
| Values represent the prevalence (%) of each Fried frailty component across acidosis persistence categories at baseline, 12 months, and 24 months. Between-group differences at each timepoint were assessed using chi-square tests. | | | | | | |

**Supplementary Table S5.** Frailty at 24 months according to acidosis persistence group and alkali treatment status

| **Group** | **n** | **Alkali treatment n (%)** | **Frail at 24 months n (%)** | **Comment** |
| --- | --- | --- | --- | --- |
| No acidosis | 68 | 0 (0.0%) | 5 (7.4%) | Reference |
| Transient — no alkali | 21 | – | 3 (14.3%) |  |
| Transient — alkali treated | 28 | 28 (100%) | 4 (14.3%) |  |
| Recurrent — no alkali | 15 | – | 3 (20.0%) |  |
| Recurrent — alkali treated | 29 | 29 (100%) | 6 (20.7%) |  |
| Persistent — no alkali | 53 | – | 12 (22.6%) | OR vs G0: 3.98 p=0.016 |
| Persistent — alkali treated | 25 | 25 (100%) | 8 (32.0%) | p (treated vs untreated)=0.401 |
| Values are n (%). OR: odds ratio versus No acidosis group (Fisher's exact test). p=0.016 for Persistent untreated vs No acidosis. p=0.401 for Persistent treated vs Persistent untreated. | | | | |

**Supplementary Table S6.** Adjusted logistic regression for frailty at 24 months including alkali treatment as covariate (Persistent acidosis vs No acidosis; n=146 complete cases)

| **Variable** | **OR** | **95% CI** | **Value** |
| --- | --- | --- | --- |
| Persistent acidosis | 1.48 | 0.17–12.95 | 0.722 |
| Alkali treatment | 0.89 | 0.15–5.24 | 0.896 |
| Baseline Fried score | 9.46 | 3.10–28.88 | <0.001 |
| Age (per year) | 1.03 | 0.94–1.13 | 0.476 |
| Male sex | 0.17 | 0.03–0.91 | 0.038 |
| eGFR (per mL/min) | 0.98 | 0.94–1.03 | 0.447 |
| Hemoglobin (per g/dL) | 1.09 | 0.83–1.45 | 0.529 |
| CCI (per point) | 1.18 | 0.75–1.85 | 0.477 |
| Reference category: No acidosis (n=68). Model adjusted for persistent acidosis, alkali treatment, baseline Fried score, age, sex, eGFR, hemoglobin, and Charlson Comorbidity Index. * p<0.05. The non-significant effect of persistent acidosis in this model reflects over-adjustment due to collinearity between baseline Fried score and acidosis group (frailty prevalence 4.4% in G0 vs 20.5% in G3 at baseline). | | | |

**Supplementary Table S7. Association between acidosis persistence and frailty at 24 months: propensity score–weighted analysis**

| **Acidosis Persistence** | **N** | **Frail at 24m, n (%)** | **Mean SIPW** | **IPW-weighted OR (95% CI)** | **p value** |
| --- | --- | --- | --- | --- | --- |
| No acidosis (0/4) | 66 | 5 (7.6%) | 0.86 | Reference | — |
| Transient (1/4) | 51 | 7 (13.7%) | 1.27 | 3.45 (0.79–15.04) | 0.100 |
| Recurrent (2/4) | 38 | 8 (21.1%) | 1.03 | 4.41 (1.27–15.28) | 0.019 |
| Persistent (≥3/4) | 77 | 22 (28.6%) | 1.08 | 4.60 (1.52–13.95) | 0.007 |
| **Per category increase** | 232 | — | — | **1.43 (1.02–2.00)** | **0.037** |

**Footnote:**
Propensity scores were estimated using a multinomial ordinal logistic model incorporating baseline eGFR, hemoglobin, CRP, Charlson Comorbidity Index, age, time since transplantation, anti-HLA antibody status, and donor type. Stabilized inverse probability weights (SIPW) were applied in a weighted binomial logistic regression model with robust standard errors (HC1).

Abbreviations: OR = odds ratio; CI = confidence interval; SIPW = stabilized inverse probability weight.

**Supplementary Table S8. Association between acidosis persistence and frailty at 24 months, stratified by baseline eGFR tertile**

| **Acidosis Persistence** | **T1: eGFR <46 (N=78) Frail, n (%)** | **T2: eGFR 46–65 (N=77) Frail, n (%)** | **T3: eGFR >65 (N=77) Frail, n (%)** | **Overall (N=232) Frail, n (%)** |
| --- | --- | --- | --- | --- |
| No acidosis | 0/7 (0.0%) | 1/20 (5.0%) | 4/39 (10.3%) | 5/66 (7.6%) |
| Transient | 3/11 (27.3%) | 2/22 (9.1%) | 2/18 (11.1%) | 7/51 (13.7%) |
| Recurrent | 6/20 (30.0%) | 2/12 (16.7%) | 0/6 (0.0%) | 8/38 (21.1%) |
| Persistent | 20/40 (50.0%) | 1/23 (4.3%) | 1/14 (7.1%) | 22/77 (28.6%) |

|  | **T1** | **T2** | **T3** | **Overall** |
| --- | --- | --- | --- | --- |
| **Trend p (Spearman)** | **0.007** | 0.961 | 0.627 | **< 0.001** |
| **Adjusted OR per category (95% CI)** | 4.67 (0.80–27.06) | † | † | 1.56 (0.80–3.07) |
| **p value** | 0.086 | — | — | 0.193 |

**Footnote:**
Adjusted models include age, sex, time since transplantation, donor type, eGFR (within stratum), hemoglobin, Charlson Comorbidity Index, CRP, and baseline Fried score.

† Model did not converge due to insufficient number of events within strata (6–7 frailty events in T2 and T3).

Abbreviations: OR = odds ratio; CI = confidence interval.

**Supplementary Table S9. Association between mean serum bicarbonate and frailty at 24 months**

| **Model** | **OR per 1 mmol/L increase** | **95% CI** | **p value** | **OR per 1 mmol/L decrease ↓** |
| --- | --- | --- | --- | --- |
| Unadjusted | 0.68 | 0.57 – 0.81 | <0.001 | 1.47 (1.24–1.75) |
| Adjusted* | 0.63 | 0.44 – 0.91 | 0.013 | 1.59 (1.10–2.29) |

**Footnote:**
*Adjusted for age, sex, time since transplantation, donor type, baseline eGFR, hemoglobin, Charlson Comorbidity Index, C-reactive protein, and baseline Fried frailty score.
N = 232.

**Supplementary Table S10. Association between nadir serum bicarbonate and frailty at 24 months**

| **Model** | **OR per 1 mmol/L increase** | **95% CI** | **p value** | **OR per 1 mmol/L decrease ↓** |
| --- | --- | --- | --- | --- |
| Unadjusted | 0.71 | 0.61 – 0.83 | <0.001 | 1.40 (1.21–1.63) |
| Adjusted* | 0.67 | 0.48 – 0.93 | 0.018 | 1.49 (1.07–2.07) |

**Footnote:**
*Adjusted for age, sex, time since transplantation, donor type, baseline eGFR, hemoglobin, Charlson Comorbidity Index, C-reactive protein, and baseline Fried frailty score.
N = 232.
Nadir bicarbonate defined as the lowest value across all four time points.

**Supplementary Table S11. Association between time-varying serum bicarbonate and longitudinal Fried frailty score (linear mixed-effects model)**

| **Predictor** | **β (per 1 mmol/L increase)** | **95% CI** | **p value** | **Interpretation** |
| --- | --- | --- | --- | --- |
| Time-varying bicarbonate (mmol/L) | −0.025 | −0.042 to −0.008 | 0.004 | Significant ✓ |
| Per 1 mmol/L decrease ↓ | +0.025 | +0.008 to +0.042 | 0.004 | Higher frailty score |

**Footnote:**
Linear mixed-effects model with random intercept per patient.
Outcome: Fried frailty score (0–5) at baseline, 12 months, and 24 months.
N = 706 observations from 238 patients.
Adjusted for visit time, age, sex, time since transplantation, donor type, hemoglobin, Charlson Comorbidity Index, and C-reactive protein.
Model fitted using maximum likelihood (REML = FALSE; optimizer: L-BFGS).

**Supplementary Table S12. Frailty at 24 months among participants non-frail at baseline**

| **Acidosis group** | **N (non-frail at baseline)** | **Frail at 24 months, n (%)** | **Unadjusted OR (vs no acidosis)** | **Adjusted OR per category (95% CI)** |
| --- | --- | --- | --- | --- |
| No acidosis | 63 | 2 (3.2%) | Reference | Reference |
| Transient | 47 | 3 (6.4%) | 2.08^a^ | — |
| Recurrent | 31 | 2 (6.5%) | 2.10^a^ | —^b^ |
| Persistent | 63 | 8 (12.7%) | 4.44^a^ | —^b^ |
| Per category increase | 204 | — | 1.59 (0.99–2.53), p=0.053 | 1.44 (0.80–2.58), p=0.221 |

**Footnote:**
Excluded: 31 participants who were frail at baseline (Fried score ≥3).
Trend test (Spearman): r = 0.140, p = 0.045.

^a^Unadjusted odds ratios compared with the no-acidosis group.
^b^Group-specific adjusted models were underpowered due to the limited number of incident frailty events (n = 15 among 205 participants); therefore, only the per-category adjusted model is presented.

Adjusted for age, sex, time since transplantation, donor type, eGFR, hemoglobin, Charlson Comorbidity Index, and C-reactive protein.

**Supplementary Table S13. Lagged models: association between prior acidosis exposure and subsequent frailty**

| **Model** | **Predictor** | **Outcome** | **Effect estimate (95% CI)** | **p value** |
| --- | --- | --- | --- | --- |
| Lagged A: Unadjusted | Acidosis visits 1–3 (0–3) | Frailty (binary) at visit 4 (24 months) | OR 1.77 (1.30–2.42) | <0.001 |
| Lagged A: Adjusted* | Acidosis visits 1–3 (0–3) | Frailty (binary) at visit 4 (24 months) | OR 1.42 (0.72–2.82) | 0.316 |
| Lagged B: Adjusted* | Acidosis visits 1–2 (0–2) | Frailty (ordinal) at visit 3 (12 months) | pOR 2.09 (1.29–3.39) | 0.003 |

Footnote:
Lagged A: N = 232.
Lagged B: N = 237 (ordinal logistic regression using proportional odds model).

*Adjusted for age, sex, time since transplantation, donor type, baseline eGFR, hemoglobin, Charlson Comorbidity Index, C-reactive protein, and baseline Fried frailty score.

Abbreviations: OR = odds ratio; pOR = proportional odds ratio.

**Supplementary Table S14. Formal mediation analysis: proportion of the association between persistent metabolic acidosis and frailty mediated through kidney function (eGFR)**

| **Effect component** | **Quantification** | **Interpretation** |
| --- | --- | --- |
| Total effect (Model 2, without time-varying eGFR) | pOR = 3.97 → ln(3.97) = 1.378 | Overall association between persistent acidosis and frailty |
| Direct effect (Model 3, with time-varying eGFR) | pOR = 3.29 → ln(3.29) = 1.191 | Association independent of kidney function |
| Indirect effect (via eGFR) | Δln = 0.187 | Portion of the association mediated or confounded by kidney function |
| Proportion mediated | 0.187 / 1.378 = **13.6%** | Approximately 14% of the association is explained by kidney function |
| Direct effect (independent of eGFR) | 1.191 / 1.378 = **86.4%** | Approximately 86% of the association remains independent of kidney function |

Proportion mediated was calculated on the log-odds scale as (ln OR_total − ln OR_direct) / ln OR_total. Estimates were derived from Table 4, Models 2 and 3 of the main manuscript. Time-varying eGFR was measured at each study visit and included as a covariate in Model 3.
